# The Ontogeny of Chicken Bursal Stromal Cells Defined by Monoclonal Antibodies

**DOI:** 10.1155/1990/51431

**Published:** 1990

**Authors:** Trevor J. Wilson, Richard L. Boyd

**Affiliations:** 1 Department of Pathology and Immunology Monash Medical School Commercial Road, Prahran Victoria 3181 Australia; 2 Division of Rheumatology/Allergy-Clinical Immunology School of Medicine, TB 192 Davis California 95616 USA

## Abstract

Molecules expressed on lymphoid stromal cells influence the differentiation of lymphocytes. We have examined the expression of stromal markers, identified by monoclonal antibodies, in the chicken bursa of Fabricius during ontogenic development. These results are also consistent with the hypothesis that medullary secretory cells are of mesenchymal origin, whereas the basement membrane-associated and some medullary epithelium are derived from the endoderm. Our results have demonstrated the complexity of bursal stromal development with determinants expressed on the adult medullary stellate cells (e.g., MUI-57 and 62) and cortical macrophages (e.g., MUI-66 and 72) detected on the early em.bryonic tunica propria (e.g., MUI-57, 66 and 72) or surface epithelium (e.g., MUI-62 and 66). In addition, we provide preliminary evidence regarding potential functions of these molecules in stem cell colonization (MUI-52), early B-cell differentiation (e.g., MUI-72), late bursal B-cell development (MUI-69 and 71) and the follicle-associated epithelium transport mechanism (MUI-61 and 73).


*Developmental Immunology*, 1990, Vol. 1, pp. 31–39
Reprints available directly from the publisher
Photocopying permitted by license only

© 1990 Harwood Academic Publishers GmbH
Printed in the United Kingdom

### The Ontogeny of Chicken Bursal Stromal Cells Defined by Monoclonal Antibodies

TREVOR J. WILSON† and RICHARD L. BOYD*

*Department of Pathology and Immunology, Monash University, Medical School, Prahran, 3181, Victoria, Australia*

Molecules expressed on lymphoid stromal cells influence the differentiation of lymphocytes. We have examined the expression of stromal markers, identified by monoclonal antibodies, in the chicken bursa of Fabricius during ontogenic development. These results are also consistent with the hypothesis that medullary secretory cells are of mesenchymal origin, whereas the basement membrane-associated and some medullary epithelium are derived from the endoderm. Our results have demonstrated the complexity of bursal stromal development with determinants expressed on the adult medullary stellate cells (e.g., MUI-57 and 62) and cortical macrophages (e.g., MUI-66 and 72) detected on the early embryonic tunica propria (e.g., MUI-57, 66 and 72) or surface epithelium (e.g., MUI-62 and 66). In addition, we provide preliminary evidence regarding potential functions of these molecules in stem cell colonization (MUI-52), early B-cell differentiation (e.g., MUI-72), late bursal B-cell development (MUI-69 and 71) and the follicle-associated epithelium transport mechanism (MUI-61 and 73).

KEYWORDS: Bursa, monoclonal antibodies, ontogeny, chicken, stroma, B cells.

### INTRODUCTION

The avian bursa of Fabricius is an epithelial lymphoid organ continuous with the cloacal epithelium. Its importance in the development of humoral immunity in birds is well established (reviewed by Glick, 1983), although the precise nature of the distinct microenvironments modulating B-cell differentiation remains poorly defined. It is clear, however, that the bursal epithelium influences the maturation of B cells. Induction of B-lineage differentiation markers on stem cells has been demonstrated in vitro by their incubation with bursal epithelial cultures or soluble factors derived from the epithelial conditioned medium (Eerola et al., 1982; Boyd et al., 1983). The bursal extract "bursopoietin" also induces such markers (Brand et al., 1983).

Bursal ontogeny has been studied by many groups, although the results have contributed more to the understanding of lymphoid development than to that of the controlling microenvironment. Thus bloodborne stem cells have been established as the progenitor cells that colonize the bursa from 8–14 days of embryogenesis, where they proliferate and differentiate into functional B lymphocytes (Moore and Owen, 1966; Le Douarin et al., 1975; Houssaint et al., 1976; Lydyard et al., 1976; Pink et al., 1985). Ontogenic analysis of the bursal stromal components could potentially reveal important cells and factors involved in this sequence. Olah et al. (1986) have identified a mesenchymal secretory cell that appears to be involved in follicle formation. In addition, molecules associated with bursal epithelium, identified by monoclonal antibodies (mAb) BEP-1 and BEP-2, have initially been detected in ontogeny at 8 days and at hatching, respectively (Houssaint et al., 1986a), which may reflect their roles in different stages of lymphoid development. The present study utilizes a panel of mAb reactive with the stromal component of the avian thymus and bursa (Boyd, Wilson, et al., 1987, 1990; Boyd, Mitrangas, et al., 1987) to examine the appearance of these microenvironmental elements during ontogeny.

*Corresponding author. Department of Pathology and Immunology, Monash Medical School, Commercial Road, Prahran, 3181, Victoria, Australia.

†Present address: Division of Rheumatology/Allergy-Clinical Immunology, School of Medicine, TB 192, Davis, California, 95616.

From our ontogenic analysis of the chicken bursa of Fabricius with these reagents, we present data in this paper that support the proposals that medullary secretory cells are derived from the mesenchyme (Olah et al., 1986), whereas medullary and basement membrane-associated epithelium (BMAE) are from endodermal origin. In addition, we have identified molecules expressed on various bursal elements that potentially modulate B-cell differentiation.

### RESULTS

The expression of the antigens recognized by the mAb panel was examined at days 10, 12, 15, and 18 of gestation in addition to immediately after hatching. The antibodies included those normally reactive with the surface epithelium (S.Ep), basement membrane (BM), BMAE, medulla, follicle-associated epithelium (FAE), interfollicular epithelium, and macrophages (MØ) (Boyd, Wilson, et al., 1987; 1990). The expression of these molecules within the developing bursa can be divided into two main groups: (1) those initially reactive with the S.Ep (Table 1) and (2) those initially detected on one or more of the other components (Table 2).

Of the 28 mAb screened, 17 were initially reactive on the S.Ep, 15 of these on 10-day embryonic bursae (Table 1). These antibodies were subsequently reactive on the epithelial buds later in ontogeny (15–18 days gestation), but react with different components of the adult bursa. For example, the antigens detected by MUI-53, 58, 59, 70, and 77 were present on the adult basement membrane and

TABLE 1
The Ontogenic Distribution of Molecules, Identified by Monoclonal Antibodies, That Are Initially Detected on the Surface Epithelium During Development of the Avian Bursa of Fabricius

| Stage initially detected[a] | Adult reactivity[a] | Monoclonal(s) (MUI-No.) | Comments[a] |
|---|---|---|---|
| 10 de | S.Ep | 80 | Weak Ep buds 12–15 de |
| | S.Ep and BM | 53, 70 | Ep buds 15–18 de |
| | S.Ep and BMAE | 58, 77 | 77-Ep buds 15–18 de |
| | FAE and BMAE | 73 | Ep buds 15 de hatch |
| | IFE | 51, 60 | FAE negative |
| | S.Ep and med Ep } Cort and med fine granules | 54, 55, 81 | 55—also under S.Ep |
| | S.Ep and med stellate | 62 | |
| | Cort and rare med MØ | 66 | & isol in 10 de TP |
| 12 de | S.Ep and BMAE | 59 | |
| | S.Ep and med Ep | 65 | & isol in 10 de TP |
| | FAE and med Ep | 61, 71 | 71—med negative until posthatching |

[a]Abbreviations: BM, basement membrane; BMAE, basement membrane-associated epithelium; cort, cortex; de, days of embryogenesis; Ep, epithelium; FAE, follicle-associated epithelium; IFE, interfollicular epithelium; isol, isolated; med, medulla; MØ, macrophagelike cells; S.Ep, surface epithelium; TP, tunica propria.

TABLE 2
The Ontogenic Distribution of Molecules, Identified by Monoclonal Antibodies, That Are Initially Detected on Cells Other Than the Surface Epithelium During Development of the Avian Bursa of Fabricius

| Initial detection of antigens: localization (stage)[a] | Adult reactivity[a] | Monoclonal (MUI-No.) | Comments[a] |
|---|---|---|---|
| Isol under S.Ep (12 de) | Med stellate cells | 67 | Isol clusters |
| Isol in S.Ep (10 de) | Med Ep and BM | 75 | & basal layer of S.Ep |
| Isol TP (10 de) | Med Ep | 64, 74 | |
| (10 de) | Cort Ly CM endo and med MØ (B-L-like) | 78 | Outer cort 15 de hatching |
| (10 de) | Ly's and isol med | 36 | |
| (12 de) | Med stellate cells | 68 | |
| (12 de) | Cort MØ | 72 | |
| Isol med (15 de) | Med stellate cells | 69 | Not all foll |
| Isol MØ in foll (15 de) | Isol MØ | 79 | Cort and interstitial |
| FAE (15 de) | Negative | 52 | Positive until posthatching |

[a]Abbreviations: BMAE, basement membrane-associated epithelium; CM, corticomedullary junction; cort, cortex; de, days of embryogenesis; endo, endothelium; Ep, epithelium; foll, follicle; FAE, follicle-associated epithelium; isol, isolated; Ly, lymphocyte; med, medulla; MØ, macrophagelike cells; S.Ep, surface epithelium; TP, tunica propria.

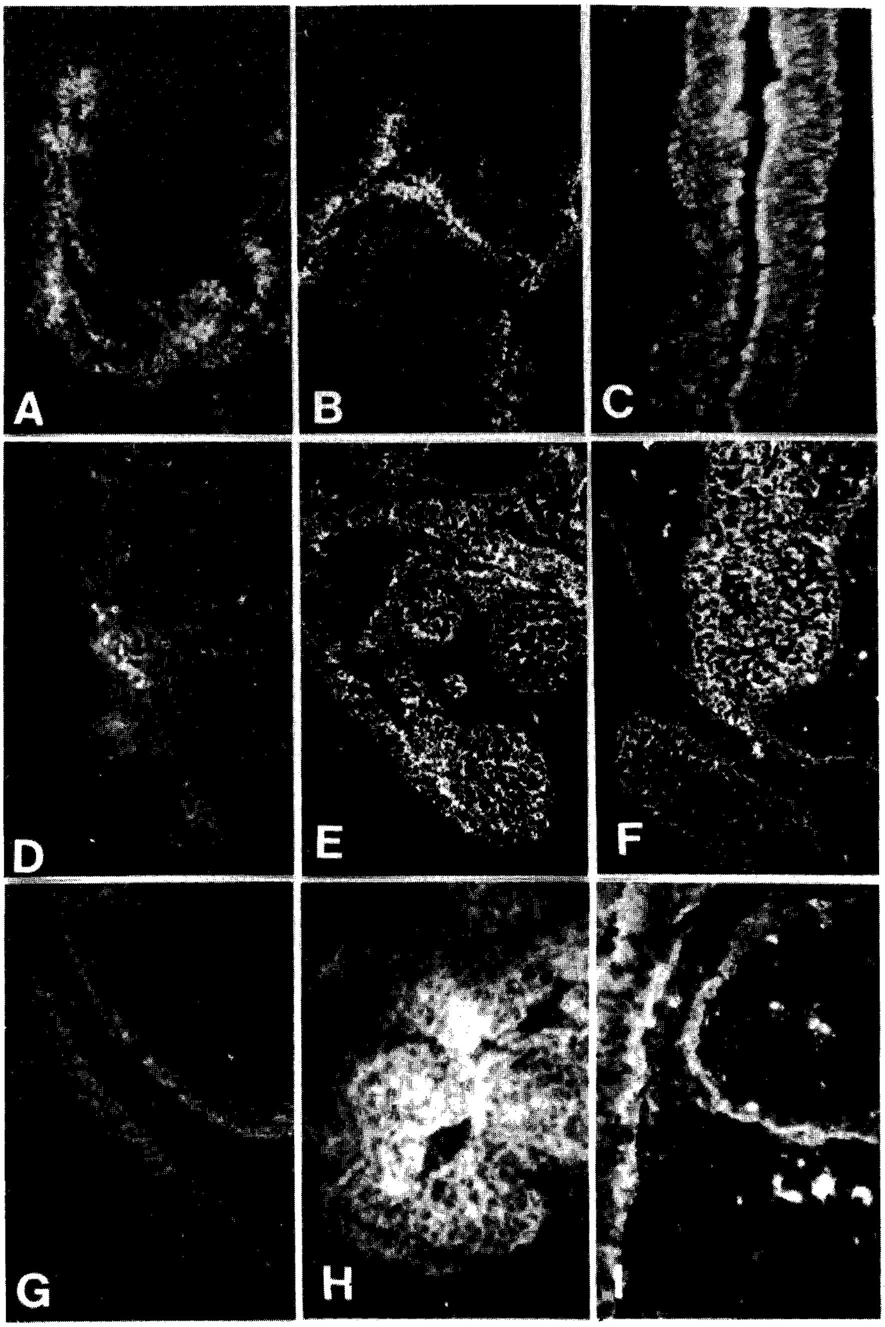

FIGURE 1. MUI-80 immunofluorescent staining of the bursal S.Ep in (A) 10 de (B) 15 de, and (C) adult (×300). MUI-75 staining of isolated cells under the S.Ep in (D) 10 de, (E) 15 de S.Ep and epithelial buds, and (F) adult bursal medulla and BM. MUI-53 staining of (G) 10 de bursal S.Ep (×120), (H) 15 de epithelial buds (×300), and (I) adult S.Ep and BM (X300). (X200 except where indicated.)

S.Ep after being detected on the early S.Ep (Fig. 1), whereas mAb MUI-54, 55, 62, 63, and 65 were also initially reactive on the S.Ep, but stain the S.Ep and medulla epithelium or stellate cells in adult birds (Fig. 2). Other markers expressed on the mature FAE (MUI-61), FAE and BMAE (MUI-73) and interfollicular epithelium (MUI-51 and 60) were present on the entire immature S.Ep, prior to the differentiation of these regions. In contrast, cells expressing the MUI-75 antigen were detected under the S.Ep at 10 days of gestation, but both the S.Ep and developing epithelial buds were positive later in ontogeny (Fig. 1). In the adult, the entire medullary epithelium but only the basal lamina layer of the S.Ep remained positive. These alterations may indicate that the S.Ep is differentiating during ontogeny.

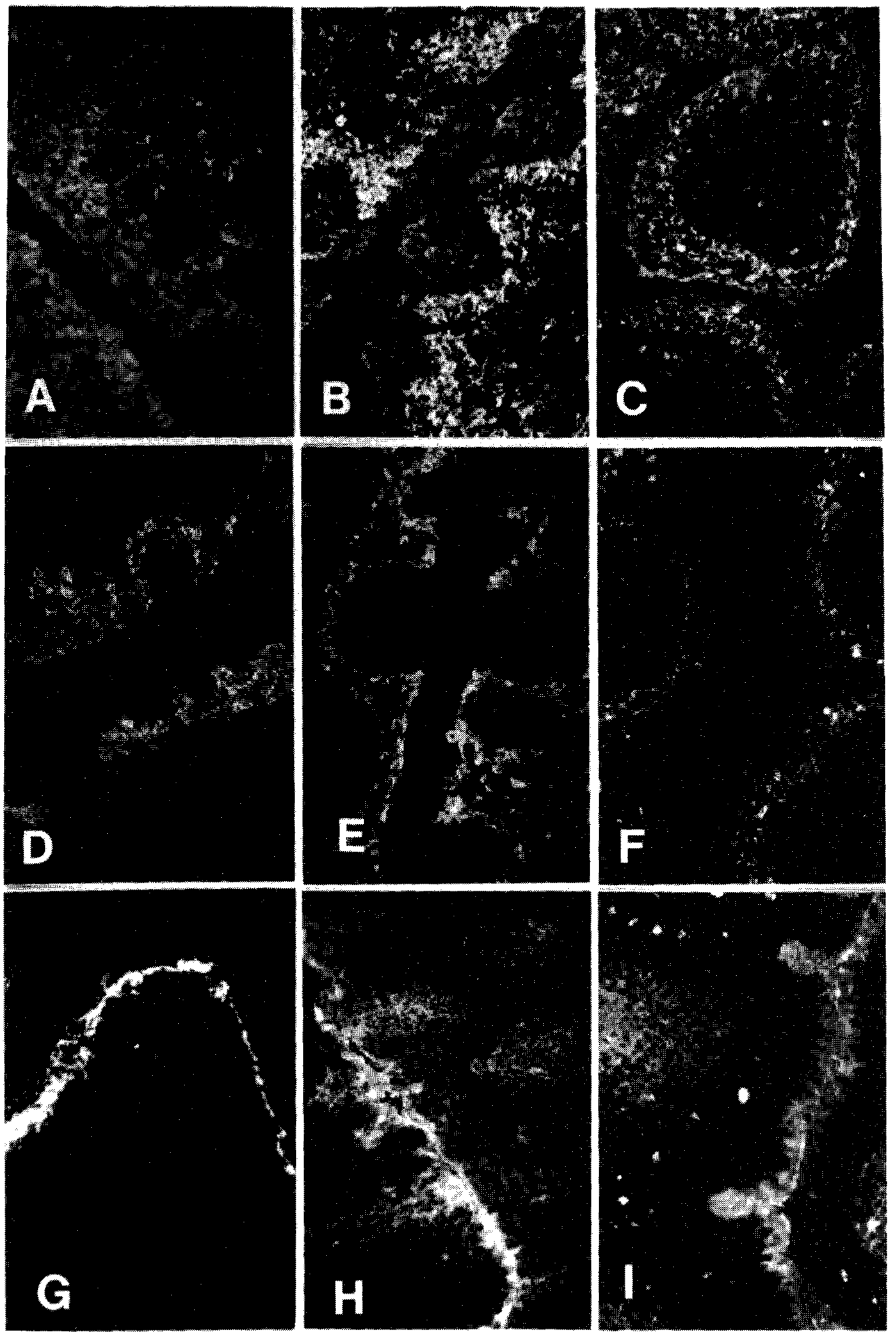

FIGURE 2. MUI-66 immunofluorescent staining of (A) 10 de S.Ep and tunica propria, (B) 15 de predominantly tunica propria, and (C) adult cortex. MUI-72 on (D) 15 de, (E) 18 de, and (F) adult bursal sections showing the progression of staining from the tunica propria to the cortex and MUI-62 demonstrating the progressive development from the (G) 12 de S.Ep to the (H) 18 de and (I) adult bursal S.Ep and medulla. (All ×200.)

In older birds, two antigens identified by MUI-66 and MUI-81 were present primarily on cortical MØ and medullary epithelium cells, respectively, with expression having been lost on the S.Ep during ontogeny (Fig. 2). This early expression may indicate an ontogenic relationship with the S.Ep, although identification of MUI-66-positive cells in the early tunica propria may be evidence for an alternative origin. Similarly, the origin of the molecule identified by MUI-53 is unclear. MUI-53 identified an antigen shared between the S.Ep and the BM. During ontogenic analysis, this epitope was initially detected at day 10 on the S.Ep, however, at day 12, isolated cells bearing this marker were identified in the tunica propria. Thus, this antigen may develop from the S.Ep, but could possibly have multiple origins within the bursa.

Many medullary antigens were detected initially at 10–12 days of incubation on isolated cells in the tunica propria, some clustered near the S.Ep (Table 2), and were observed within the developing follicles during gestation. The majority of antigens that appear to develop from the S.Ep were also found on the epithelial buds between day 15 of embryogenesis and hatching. These included molecules that are expressed on the adult S.Ep (MUI-80) (Fig. 1), BM (MUI-53 and 70), BMAE (MUI-77), BMAE and FAE (MUI-73), FAE (MUI-61), medullary epithelium (MUI-54 and 55), and medullary stellate cells (MUI-62 and 63) (Fig. 3). MUI-51 and 60 were S.Ep antigens that were expressed on the interfollicular epithelium, but not on the FAE or the epithelial buds. In the 10-day embryonic bursae, the MUI-60 antigen was expressed on some, but not all of the S.Ep, whereas MUI-51 was expressed on the entire S.Ep. Regions of the S.Ep, therefore, may undergo some differentiation prior to the formation of epithelial buds.

Molecules on the FAE, identified by MUI-52 (Fig. 4), were initially expressed concomitant with bud generation and the development of the FAE transport mechanism (Beezhold et al., 1983). Curiously, MUI-52 was only expressed until hatching in the bursa, being detected only on isolated cortical

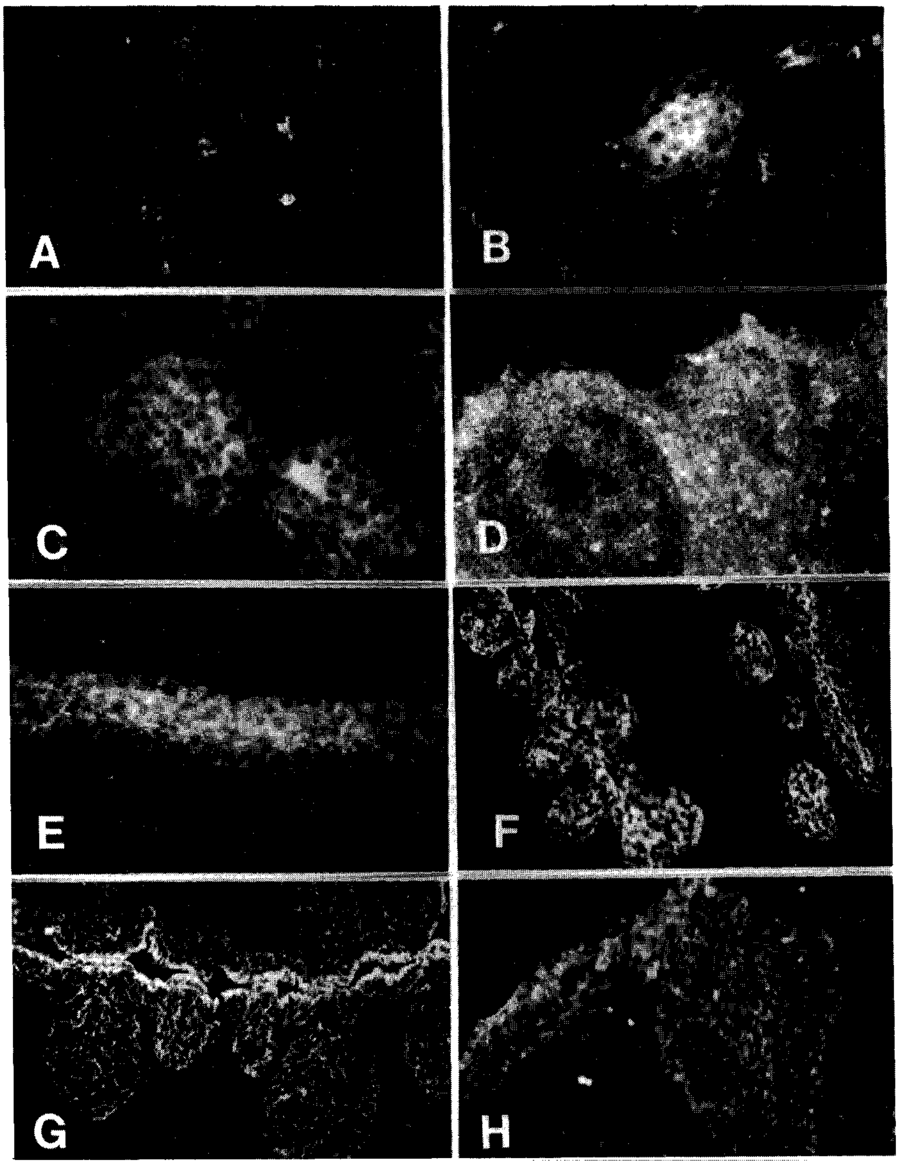

FIGURE 3. MUI-57 immunofluorescent staining on (A) 10 de, (B) 12 de, (C) 15 de (×300), and (D) adult (×120) bursae showing the progression of this antigen from isolated cells to the entire follicle. The development of the MUI-55 antigen was from S.Ep in the (E) 10 de (×300) to the medulla and S.Ep in the (F) 15 de, (G) 18 de, and (H) adult bursae. (×200 except where indicated.)

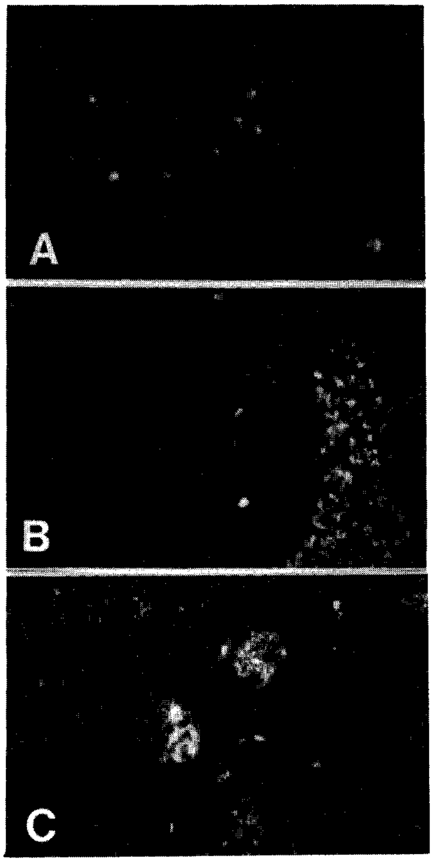

FIGURE 4. MUI-69 immunofluorescent staining of isolated cells in the (A) 15 de tunica propria, and medullary cells of some follicles in the (B) adult (X 120). MUI-52 staining of the FAE in the (C) 18 de. (×200).

thymic epithelial cells in the normal adult chicken. The antigen detected by mAb MUI-71, however, was not expressed at adult levels on the FAE until posthatching. This marker was initially detected at low level on the 10-day embryonic S.Ep and showed no alteration during gestation. Posthatching this antigen appeared in the medulla and on the FAE, whereas its expression was lost on the interfollicular epithelium.

MUI-69 identified a medullary molecule on non-epithelial cells in the adult, which first appeared in the tunica propria of the 15-day embryo (Fig. 4). Throughout ontogeny, cells expressing this marker were not detected in all follicles.

### DISCUSSION

This analysis of the ontogenic development of the bursa of Fabricius suggests that the bursal microenvironmental elements are also derived from complex interactions between the precursor cell types, rather than only via a sequential differentiation process. Thus, from these results, we propose that these stromal components are predominantly progeny of the cells of the S.Ep (endoderm) and tunica propria (mesenchyme) (Le Douarin et al., 1975), differentiating following interaction between these structures. Many of the components are not mutually exclusive with respect to their origin, with different antigens expressed on the medullary epithelium, medullary stellate cells, BMAE and cortical MØ initially being detected on either the tunica propria or S.Ep.

The importance of the S.Ep in the development of the bursal microenvironmental components is indicated by the number of distinct molecules expressed on the early S.Ep that are present on various components of the mature bursa. Whether this expression means that these components differentiate from, acquire antigens from, or are activated by the S.Ep has not been determined. Construction of quail-chick chimeric bursae has demonstrated that the S.Ep, BMAE, and the medullary epithelium are derived from the endoderm, whereas the interfollicular connective tissue cells are of mesodermal origin (Le Douarin et al., 1975; Houssaint et al., 1976). These results correspond to the staining of many of the mAb, e.g., MUI-79, which stained isolated cells in the tunica propria initially, before being reactive on interstitial MØ in the adult and MUI-65, an antibody that initially detects the S.Ep in the 10-day embryo and stains the S.Ep and medullary epithelium in the adult. Similar to a number of other mAb, MUI-65 also detected isolated cells in the tunica propria (mesenchyme) early in ontogeny. It is therefore possible that some bursal antigens are not restricted to expression on only endodermal or mesenchymal cells. Conversely, three other mAb also stained the medullary epithelium after being initially reactive on cells in the tunica propria. Whether these molecules are expressed on the same multipotential cell or on different mesenchymal cell types is unclear. The medullary region may thus contain epithelial cell populations derived from both endoderm and mesoderm, perhaps some form of hybrid cells. From our results, however, it appears improbable that it contains epithelium exclusively derived from the endoderm.

It also seems likely that there is an interaction between the endodermal and mesodermal cells prior to the formation of epithelial buds. Olah et al. (1986)

observed that differentiated "dark" mesenchymal cells migrated to and entered the S.Ep before the induction of the epithelial buds. Our experiments have demonstrated MUI-67-positive cells clustered under the S.Ep and cells expressing MUI-75 within the S.Ep of young embryos. These antibodies also stain the adult bursal medulla, supporting Olah's proposal that this interactive mesenchymal cell is a precursor for medullary secretory cells (Olah et al., 1986). Subsequent to, and possibly as a result of, this interaction, the FAE and epithelial buds are formed. Houssaint and Hallet (1986) established that the FAE consisted of modified epithelial cells and of haemopoietic cells. Our data indicate that the FAE is primarily derived from cells of epithelial (endodermal) origin since thirteen antigens initially expressed on the S.Ep were present on cells of the epithelial buds and FAE during ontogeny. Many of these molecules were lost late in ontogeny, although six were still expressed in the adult chicken. The loss of these markers suggests that alterations in the epithelium are occurring as the FAE differentiates into a functional and antigenically distinct region. The transient expression of some molecules (e.g., the antigen detected by MUI-52) on the FAE from day 15 until hatching corresponds chronologically to the recruitment of lymphoid precursors and their subsequent localization within the lymphoid follicles (Houssaint et al., 1976; Ratcliffe et al., 1987). The MUI-52 antigen is also re-expressed in neonatally cyclophosphamide-treated birds (Wilson and Boyd, 1990). The other modifications of the FAE possibly involve molecules that have a function in the FAE transport mechanism (e.g., MUI-61 and 73). This is the means by which material from the bursal lumen is pinocytosed by the cells of the FAE and transported into the follicles (Bockman and Cooper, 1973; Beezhold et al., 1983), where it presumably stimulates a specific B-cell response (Van Alten and Meuwissen, 1972). The current investigation of these molecules could provide important evidence regarding the control of B-cell differentiation by these stromal components.

Haemopoietic stem cells seed the developing embryonic bursa between days 8 and 14 of gestation (Le Douarin et al., 1975), possibly via adherence to the endothelium of blood vessels located in close proximity to the bursal epithelium (Le Douarin, 1986). It has been postulated that a molecule secreted by the epithelium is involved in the chemoattraction of these precursors (Le Douarin, 1978) and haemopoietic activity has also been observed in the early developing bursa (Ackerman and Knouff, 1964). Potentially, these molecules may be among those identified by the mAb reactive with the early epithelium. The precursors then localize in the tunica propria, where induction of their differentiation into B cells may be initiated (Boyd and Ward, 1978). It is possible mAb MUI-57, 64, 68, 72, 74, and 78 identify stromal elements involved in this process as they recognize nonlymphoid cells in the tunica propria early in ontogeny. MUI-72 also stains MØ in the adult bursal cortex, the more immature region of the bursa (Boyd and Ward, 1984), whereas the identification of the other markers (MUI-57 and 75) in the embryonic cortex may be evidence of having functions related to early steps in B-lymphocyte differentiation.

Similarly, molecules that appear in the bursal medulla later in ontogeny (e.g., MUI-69 and 71) are more likely to be involved in the latter stages of B-cell differentiation. MUI-69 was not expressed on all follicles, which may relate to the observation that cyclophosphamide-treated, lymphoid reconstituted bursae have follicles that are either clonally repopulated or empty (Sorvari et al., 1974; Pink et al., 1985). In addition, the molecules that are lost as the bird matures may possess a function that is only expressed early; e.g., rearrangement of the chicken Ig genes has been shown to occur only during ontogenic development (reviewed by Weill and Reynaud, 1987).

The ontogenic expression of the bursal stromal antigens in this study concurs with earlier proposals regarding the mesenchymal origin of bursal medullary secretory cells (Olah et al., 1986) and the endodermal origin of the BMAE and medullary epithelium (Le Douarin et al., 1975; Houssaint et al., 1976), although our data indicate that the medullary epithelium probably results from an interaction between endodermal and mesenchymal cells. We have also provided preliminary evidence identifying potentially important molecules involved in the bursal recruitment of precursor cells and the mechanisms controlling B-lymphocyte differentiation. These molecules have also been examined in birds with humoral immunodeficiency induced by testosterone propionate or cyclophosphamide (Wilson and Boyd, 1990). This comparative analysis has provided further insight into the potential function of these antigens. Further experimentation regarding the functions of these markers and their characterization is currently in progress.

### MATERIALS AND METHODS

### Animals

Australorp ×White Leghorn F1 hybrid chicken embryos were obtained from Research Poultry Farm (Research, Victoria, Australia) and incubated in a humidified incubator (Multiplo) at 39°C.

### Tissue Sections

Chick embryos were killed (six per group) at 10, 12, 15, and 18 days of incubation and immediately post-hatching. Bursae, spleens, and thymuses were removed, immersed in Tissue-Tek (Miles Scientific) and snap-frozen on a liquid nitrogen-isopentane slurry.

### Monoclonal Antibodies

A panel of mAb reactive with the avian bursal and thymic stroma was used in this study. It was prepared and characterized as described elsewhere (Boyd, Wilson, et al.; 1987; 1990).

### Indirect Immunofluorescence

Cryostat sections (4 $\mu$m) were incubated with culture supernatant from the hybridoma or NS-1 myeloma cells (20 min), washed three times with PBS (1×1 min, 2×5 min), incubated with FITC-conjugated sheep antimouse immunoglobulin (1/100, Silenus Labs, Melbourne, Australia) for 20 min and washed as before. Sections were mounted using veronal buffered glycerol or Permafluor (Lipshaw, Detroit, Michigan) and examined using either a Leitz Diavert fluorescence or a Zeiss epifluorescence microscope or a Zeiss MC63 camera and Kodak Ektachrome 1600 ASA film were used for photography. Double labeling with rabbit antihuman keratin (Dako, Santa Barbara, California) and sheep antirabbit-rhodamine (1/50, Silenus) was also performed to enable recognition of epithelial cells.

### ACKNOWLEDGMENTS

This work was supported by grants from the Australian National Health and Medical Research Council (grant No. 860396) and the Anti-cancer Council of Victoria (grant No. 131658).

*(Accepted December 1, 1989)*

### REFERENCES

Ackerman G.A., and Knouff R.A. (1964). Lymphocytopoietic activity in the bursa of Fabricius. *In:* The thymus in immunobiology, Good R.A., and Gabrielsen A.E., Eds. (New York: Harper and Row), pp. 123–149.

Beezhold D.H., Sachs H.G., and Van Alten P.J. (1983). The development of transport ability by embryonic follicle-associated epithelium. J. Retic. Soc. **34:** 143–152.

Bockman D.E., and Cooper, M.A. (1973). Pinocytosis by epithelium associated with lymphoid follicles in the bursa of Fabricius, appendix and Peyers patches. An electron microscopic study. Am. J. Anat. **136:** 455–478.

Boyd, R.L., Mitrangas K., Ramm H.C., Wilson T.J., Fahey K.J. and Ward H.A. (1987). Chicken B lymphocyte differentiation: ontogeny, bursal microenvironment and effect of IBD virus. Prog. Clin. Biol. Res. **238:** 41–51.

Boyd R.L., and Ward H.A. (1978). Lymphoid antigenic determinants of the chicken: cellular representation and tissue localization. Immunology **34:** 9–17.

Boyd R.L., and Ward H.A. (1984). Lymphoid antigenic determinants of the chicken: ontogeny of bursal dependent lymphoid tissue. Dev. Comp. Immunol. **8:** 149–167.

Boyd R.L., Ward H.A., and Muller H.K. (1983). Bursal and thymic epithelial cells in the chicken: induction of B- and T-lymphocyte differentiation by in vitro monolayer cultures. J. Retic. Soc. **34:** 383–393.

Boyd R.L., Wilson T.J., Mitrangas K., and Ward H.A. (1987). Characterization of chicken thymic and bursal stromal cells. Prog. Clin. Biol. Res. **238:** 29–39.

Boyd R.L., Wilson T.J., Ward H.A. and Mitrangas K. 1990. Phenypic characterization of chicken bursal stromal elements. Dev. Immunol. (*in press*).

Brand A., Galton J., and Gilmour D.G. (1983). Committed precursors of B and T lymphocytes in chick embryo bursa of Fabricius, thymus and bone marrow. Eur J. Immunol. **13:** 449–455.

Eerola E., Lassila O., Gilmour G., and Toivanen A. (1982). Induction of B-cell differentiation in vitro by bursal epithelium. J. Immunol. **128:** 2652–2655.

Glick B. (1983). Bursa of Fabricius. *In:* Avian biology VII, Farner D.S., King J.R. and Parkes K.C. Eds. (New York: Academic Press), pp. 443–500.

Houssaint E., Belo M., and Le Douarin N.M. (1976). Investigations of cell lineage and tissue interactions in the developing bursa of Fabricius through interspecific chimeras. Dev. Biol. **53:** 250–264.

Houssaint E., Diez E., and Hallet M.M. (1986). The bursal microenvironment: phenotypic characterization of the epithelial component of the bursa of Fabricius with the use of monoclonal antibodies. Immunol. **58:** 43–49.

Houssaint E., and Hallet M.M. (1986). The follicle-associated epithelium in the bursa of Fabricius. Cell origin studied by means of quail-chick chimeras and monoclonal antibodies. J. Leuk. Biol. **40:** 469–477.

Le Douarin N.M. (1978). Ontogeny of haematopoietic organs studied in avian embryo interspecific chimeras. *In:* Differentiation of normal and neoplastic haematopoietic cells, Clarkson B., Marks P.A. and Till J.E., Eds. (Cold Spring Harbor, NY: Cold Spring Harbor Laboratory), pp. 5–31.

Le Douarin N.M. (1986). The microenvironment of T and B lymphocyte differentiation in avian embryos. Cur. Top. Dev. Biol. **20:** 291–313.

Le Douarin N.M., Houssaint E., Jotereau F.V., and Belo M. (1975). Origin of haemopoietic stem cells in embryonic bursa of Fabricius and bone marrow studied through interspecific chimeras. Proc. Natl. Acad. Sci. USA **72:** 2701–2705.

Lydyard P.M., Grossi C.E., and Cooper M.D. (1976). Ontogeny of B-cells in the chicken. I. Sequential development of clonal diversity in the bursa. J. Exp. Med. **144:** 79–97.

Moore M.A.S., and Owen, J.J.T. (1966). Experimental studies on the development of the bursa of Fabricius. Dev. Biol. **14**: 40–51.

Olah I., Glick B., and Toro I. (1986). Bursal development in normal and testosterone-treated chick embryos. Poult. Sci. **65**: 574–588.

Pink J.R., Vainio O., and Rijnbeck A.M. (1985). Clones of B-lymphocytes in individual follicles of the bursa of Fabricius. Eur. J. Immunol. **15**: 83–87.

Ratcliffe M.J.H., Lassila O., Reynolds J., Pink J.R.L., and Vainio O. (1987). A reevaluation of the function of the bursa of Fabricius. Prog. Clin. Biol. Res. **238**: 3–14.

Sorvari T., Toivanen A., and Toivanen P. (1974). Transplantation of bursal stem cells into cyclophosphamide treated chicks. Transplantation **17**: 584–592.

Van Alten P.J., and Meuwissen H.J. (1972). Production of specific antibody by lymphocytes of the bursa of Fabricius. Science **176**: 45–47.

Weill J.-C., and Reynaud C.-A. (1987). The chicken B cell compartment. Science **238**: 1094–1098.

Wilson T.J., Mitrangas K., Ramm H.C., Boyd R.L., and Ward H.A. (1988). Response of the chicken bursal stroma to treatment with cyclophosphamide and IBD virus. Adv. Exp. Med. Biol. **267**: 75–80.

Wilson T.J. and Boyd R.L. (1990). Cyclophosphamide and testosterone—induced alterations in chicken bursal stroma identified by monoclonal antibodies. Immunology (in press).

